# Urban mosquitoes and filamentous green algae: their biomonitoring role in heavy metal pollution in open drainage channels in Nairobi industrial area, Kenya

**DOI:** 10.1186/s12862-021-01913-7

**Published:** 2021-10-11

**Authors:** Geoffrey Kariuki Kinuthia, Veronica Ngure, Luna Kamau

**Affiliations:** 1 Department of Science, Engineering & Health, Daystar University, PO Box 44400 - 00100 GPO, Nairobi, Kenya; 2 Department of Biological Sciences, Laikipia University, PO Box 1100 – 20300, Nyahururu, Kenya; 3 Center for Biotechnology Research and Development (Malaria laboratory), Kenya Medical Research Institute (KEMRI), PO Box 548840 – 00200, Nairobi, Kenya

**Keywords:** Heavy metals, Wastewater, Open channels, Urban mosquitoes, Filamentous algae, Biomonitoring

## Abstract

**Background:**

Industrial wastewater is a human health hazard upon exposure. Aquatic organisms in contaminated wastewater may accumulate the toxic elements with time. Human population living in informal settlements in Nairobi industrial area risk exposure to such toxic elements. Biomonitoring using aquatic organisms in open drainage channels can be key in metal exposure assessment. Levels of Mercury (Hg), Lead (Pb), Chromium (Cr), Cadmium (Cd), Thallium (Tl), and Nickel (Ni) were established in samples of wastewater, filamentous green algae (*Spirogyra*) and mosquitoes obtained from open drainage channels in Nairobi industrial area, Kenya.

**Results:**

Pb, Cr, & Ni levels ranged from 3.08 to 15.31 µg/l while Tl, Hg, & Cd ranged from 0.05 to 0.12 µg/l in wastewater. The Pb, Cr, Ni, & Cd levels were above WHO, Kenya & US EPA limits for wastewater but Hg was not. Pb, Cr, Tl, & Ni levels in assorted field mosquitoes were 1.3–2.4 times higher than in assorted laboratory-reared mosquitoes. Hg & Cd concentrations in laboratory-reared mosquitoes (0.26 mg/kg & 1.8 mg/kg respectively) were higher than in field mosquitoes (0.048 mg/kg & 0.12 mg/kg respectively). The levels of Pb, Cr, & Ni were distinctively higher in field mosquito samples than in wastewater samples from the same site. Pb, Cr, Ni, Cd & Hg levels in green filamentous *Spirogyra* algae were 110.62, 29.75, 14.45, 0.44, & 0.057 mg/kg respectively. Correlation for Pb & Hg (r (2) = 0.957; P < 0.05); Cd & Cr (r (2) = 0.985; P < 0.05) in algae samples was noted. The metal concentrations in the samples analyzed were highest in filamentous green algae and least in wastewater.

**Conclusion:**

Wastewater, mosquitoes, and filamentous green algae from open drainage channels and immediate vicinity, in Nairobi industrial area (Kenya) contained Hg, Pb, Cr, Cd, Tl, and Ni. Mosquitoes in urban areas and filamentous green algae in open drainage channels can play a role of metal biomonitoring in wastewater. The potential of urban mosquitoes transferring heavy metals to human population from the contaminated wastewater should be investigated.

## Background

The wide application of heavy metals has raised concerns over their potential harmful effects on human health and environmental modification [1]. Environmental pollution by heavy metals has been associated with mining, foundries, smelters, and other metal-based industrial operations [2]. Harmful health effects associated with heavy metals in exposed humans and animals range from cancer, systems disorders, developmental anomalies, neurologic and neuro-behavioral disorders, hematologic disorders, DNA damage, cellular and tissue damage, and gastrointestinal toxicity [3–7]. According to Tchounwou et al. [1], heavy metals toxicity depends on their dose, route of exposure, chemical property as well as age, gender, genetics, and nutritional status of the exposed individuals.

Biological monitoring of water quality involves use of aquatic organisms to measure level of exposure to various pollutants [8]. For instance, heavy metals have previously been reported in mosquito larvae [9]. Biomonitoring of aquatic pollutants using *Culex* mosquito larvae is advantageous because their larvae are able to survive in polluted water and secondly, the *Culex* larvae proliferate fast and have a sufficient developmental interval which gives time for heavy metal uptake [10–12]. Previous survey of mosquitoes in Nairobi industrial area, showed that 95% of the trapped adult mosquitoes were of the species *Culex pipiens* [13]*.* Mosquitoes found in urban areas have been reported to adapt to changing environments by being able to breed in polluted waters. The adaptation requires the continuous substitution of new beneficial alleles at a rate that is proportional to the rate of environmental change [14].

According to Kitvatanachai et al. [12], the routine collection of urban mosquitoes for medical research can also avail appropriate samples for monitoring environmental pollution by heavy metals. The uptake of pollutant metals by the mosquito larvae inhabiting contaminated water may occur through direct body absorption or indirectly through ingesting heavy metals contaminated materials. While adult mosquitoes suck nectar, honey and animal blood, their larvae filter algae and other plant materials from the water. According to Tuno et al. [15], algae are important food resources of the African mosquito larvae. However, the larvae of *Toxorhynchites* mosquitoes are predacious and feed on the larvae of other mosquito species, but in absence of a suitable prey they may feed on detritus or exhibit cannibalism [16].

According to Marten [17], abundance of algae usually provides favorable conditions for mosquito proliferation. *Spirogyra* filamentous algae which usually forms mats in the water serve as mosquito larvae food [18–20]. Some species of algae however, including those in the order Chlorococcales and the blue green algae (Cyanobacteria) have larvicidal effect because they are indigestible and toxic to mosquito larvae respectively [17]. There are about 200 species of green algae that cannot be digested by mosquito larvae [21]. Hexane and chloroform extracts from marine Phaeophyta algae (*Padina gymnospora*) have been reported to display larvicidal activity against *Aedes aegypti* [22].

Certain species of algae have been reported to take up heavy metals from contaminated water through biosorption and bioaccumulation [23]. Such species can therefore be used as indicators of the extent of water pollution and in removing pollutants from the wastewater, a process known as phytoremediation. Phytoremediation has emerged as a desirable technology which uses plants for removal of contaminants from water or soil [24]. Both micro and macro algae have been shown to take up heavy metals from contaminated water naturally and from experimental solutions in the laboratory [25, 26]. Aquatic organisms in the lower trophic levels are better tools for natural biomonitoring of metal since they are among the first in the food chain to be exposed to the pollutants [27]. The heavy metals taken up by the aquatic producers flow into the consumers in a food web through the various aquatic food chains. Use of algae for environmental biomonitoring can be advantageous because they are relatively common and develop spatially dense populations and are easy to sample [28].

Drainage channels with wastewater create aquatic ecosystems which supports populations of different organisms that may include filamentous organisms, insects, arachnids, microorganisms, higher plants, amphibians, fish, among others. Slow flowing wastewater tend to support diverse species of organisms. Wastewaters in industrial areas are often contaminated with toxic heavy metals [29]. Therefore, there is a possibility of the toxic heavy metals accumulating in the tissues of the aquatic organisms with time. The aquatic green plants in open drainage channels may absorb the contaminants present in the wastewater directly through their surfaces and roots, while the consumers may do so through direct surface absorption and feeding processes. Establishing the levels of contaminants in the wastewater, algae (producers) and mosquitoes (consumers) can validate the possibility of a biomonitoring role of the organisms.

The current study was therefore designed to establish the levels of selected heavy metals in samples of wastewater, filamentous algae (Order Zygnematales: Genus *Spirogyra*) and mosquitoes (Order Diptera: Family Culicidae) both larvae and adults, that were obtained from open drainage channels and the immediate vicinity in Nairobi industrial area, Kenya. The metallic elements studied were chromium (Cr), cadmium (Cd), mercury (Hg), lead (Pb), nickel (Ni), and thallium (Tl). The metals selected for the current study have a high degree of toxicity and rank among priority metals that are of public health concern [1] and that some of the selected metals including Cd, Cr, Pb and Ni are commonly found in contaminated wastewater [30].

## Methods

### Study area and sampling sites

**Fig. 1:**
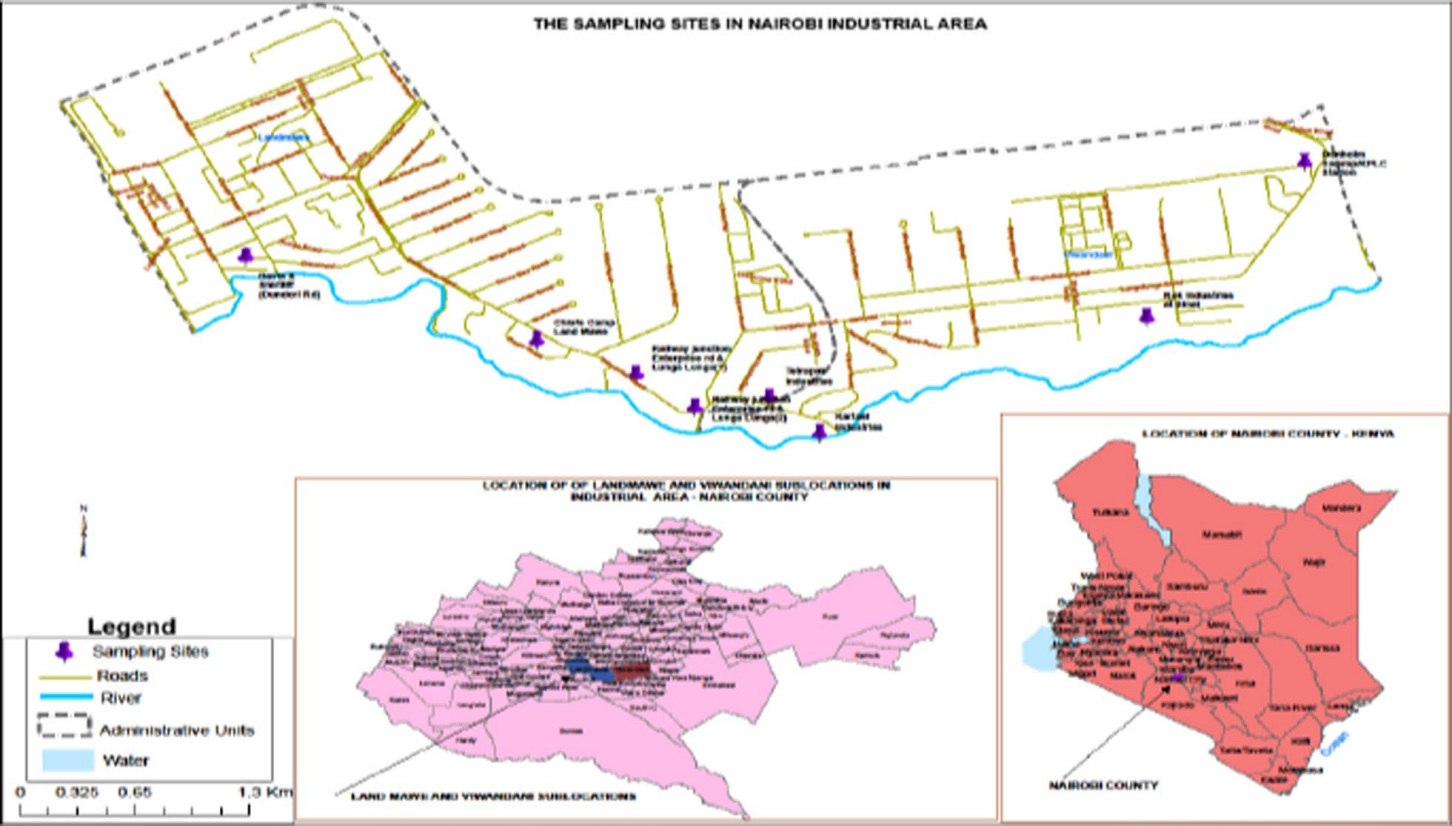
The study area and the sampling sites in Nairobi industrial area in Kenya ( Source: Kenya National Bureau of Statistics (KNBS); The map was drawn using Software ArcMap Version 10.61)

Nairobi River is a tributary of Athi River, which then flows into the Indian Ocean [31]. One of the tributaries of Nairobi River, is Ngong River that passes through Nairobi industrial area, where the current study was undertaken (Fig. 1). Along the Ngong river are the Mukuru and Viwandani slums and the many villages that constitute them. These villages spread within and on the periphery of Nairobi industrial area. Samples of wastewater, filamentous algae and mosquito larvae were obtained from main open drainage channels that were directly or indirectly draining into the Ngong river. The sampling sites were randomly selected near the main roads for easy accessibility. Adult mosquitoes were trapped at night from the factory premises (for the security of the traps) near the open drainage channels selected for the current study. The sampling was carried out over a period of 30 days in the month of August, which is a relatively dry period that extends from June to October [32]. Samples were collected from eight different sampling sites that were coded A to H, at Nairobi industrial area, Kenya. The sites included Tetrapak (A); Chief’s Camp at Land Mawe (B); Two sites at Railways near Enterprise/Lunga Lunga roads junction (C & D); Davis & Shirtliff along Dondori road (E); Kartasi Industries (F); Rok Industries near Sinai village in Viwandani slum (G); and Donholm Swamp/Kenya Power & Lighting Station (H) as shown in Fig. 1. The permit to carry out the current research was awarded by the National Commission for Science, Technology, and Innovation (NACOSTI) through a Research Clearance Permit No. NACOSTI/P/15/8787/5184. Further authorization was awarded by Nairobi County Commissioner, Nairobi County Director of Education, Deputy County Commissioner in Starehe Sub-County, Deputy County Commissioner for Makadara Sub-County, and from Daystar University (CRPCPB/14/7/0015).

### Collection and preparation of wastewater samples

A standard 350 mL dipper was used to collect the wastewater samples from the open channels and placed into clean reagent plastic bottles. The samples were collected in triplicates in equal portions. Two separate portions were separately acidified with concentrated hydrochloric (HCL) acid and concentrated nitric (HNO_3_) acid respectively by adding three drops of the respective acid per 100 ml of wastewater sample. Acidification was meant to inhibit adsorption of dissolved elements onto the interior walls of the plastic bottles as well as preventing microbial reactions [33]. A third portion of wastewater was not acidified to act as a control. All the samples were labeled appropriately, packaged, and stored in low temperature.

### Measuring the physico-chemical parameters of water samples

The physico-chemical parameters of the wastewater samples including temperature, pH, electrical conductivity (EC), and total dissolved solids (TDS) were measured immediately after collection of wastewater samples at the site using a digital electronic device (HANNA Instruments, H1991300, Romania) and recorded appropriately.

### Collecting samples of filamentous green algae

**Fig. 2:**
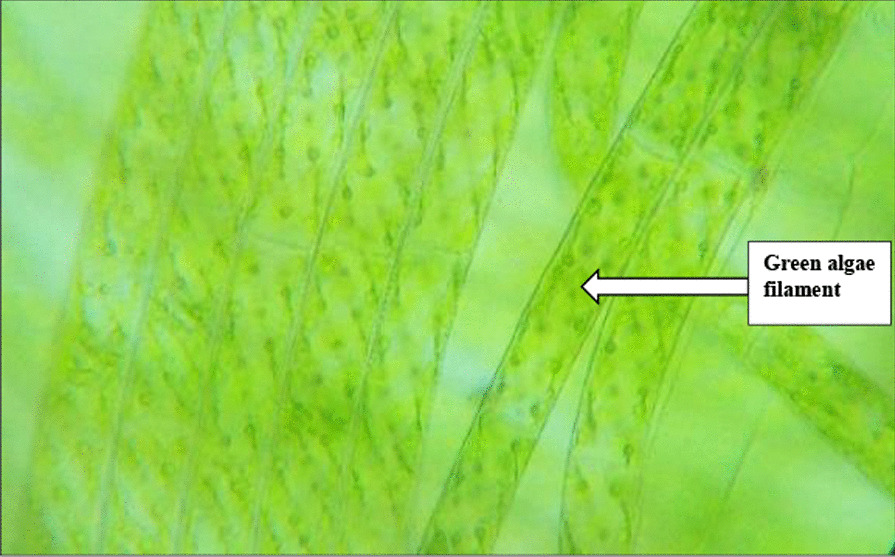
Filamentous green algae (Genus *Spirogyra*) collected from wastewater in open drainage channels near Kartasi industries, Nairobi

Filamentous green algae (Fig. 2) were collected in triplicates from wastewater in open drainage channels using a large plastic strainer and packaged in well labeled brown paper bags. The strainer was then rinsed in deionized water before being used again. All the samples collected were transferred to Kenyatta University Biochemistry laboratory for identification and further processing. The genus *Spirogyra* of the filamentous green algae (Fig. 2) was morphologically identified by the authors and support researchers based on the algae’s notable spiral chloroplasts and unbranched filamentous strands.

### Preparation of green algae samples for heavy metal analysis

The filamentous green algae samples were divided into two parts. One part was air dried at room temperature for several days while the remaining part was lyophilized (freeze dried). Both air dried and lyophilized algae samples were ground and sieved to obtain a fine powder as described by Ngure and Kinuthia [34]. The powder was then weighed and packaged in well labeled brown small envelops to await metal analysis. Briefly, lyophilization involved extracting the algae samples using de-ionized water for 36 h on an electrical shaker, followed by filtering the extract obtained using clean muslin cloth on a water pump. About 200 ml of the filtrate was then put on clean stainless-steel tray and placed in the deep freezer for 24 h at negative 45 °C. The samples were then retrieved and placed in a freeze-drier for a further 24 h at negative 50 °C to complete lyophilization.

### Outdoor trapping of adult mosquitoes

**Fig. 3:**
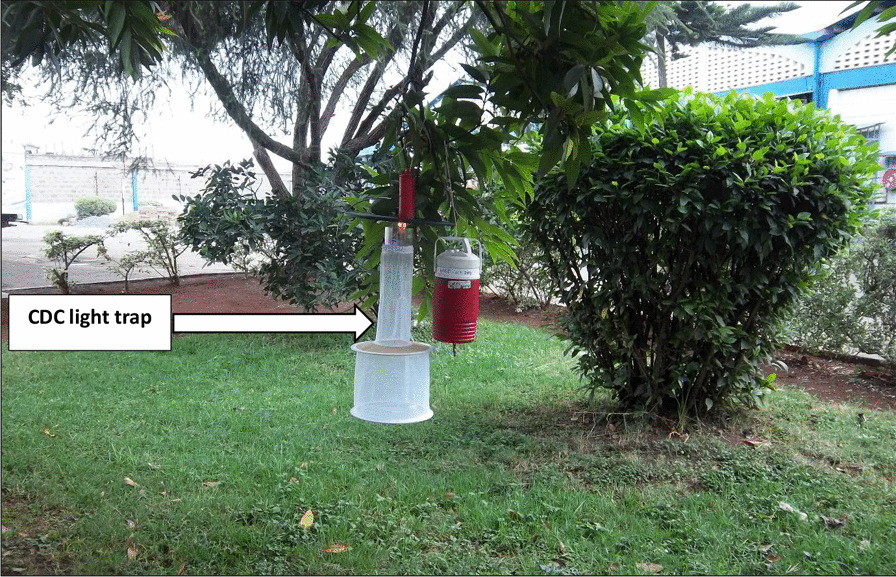
CDC mosquito trap on a tree branch in the premise of Kartasi Industries, Nairobi

Adult mosquitoes were trapped using surveillance standard Centers for Disease Control and prevention (CDC) light traps as described by Mweya et al. [35] using carbonated dry ice as the bait. The traps were set in potential breeding sites and amidst the vegetation where applicable (Fig. 3) within the factory premise. The trapping commenced from 6:00 PM to 6:00 AM each day. The average number of CDC traps set per sampling site per night was seven depending on the size of the compound. The mosquito trapping activity was carried out daily for 2 weeks. The field mosquitoes were trapped near the sites where wastewater and algae samples had been collected from.

### Collection of mosquito larvae from wastewater

Mosquito larvae were collected during the day preferably midmorning, from wastewater in open drainage channels. Three dips (triplicate) were taken to obtain the larvae from the wastewater, using the standard 350 mL dipper. If less than ten mosquito larvae were captured in the first three attempts, additional two dips were done to obtain a sizable number. The dipper contents were then transferred onto a white plastic tray. The mosquito larvae were sorted, counted and their number per dip per site recorded. The larvae were then placed in plastic Whirl–Pak® bags (Bio Quip, Rancho Dominguez, CA) which were approximately half full of the same wastewater from which the larvae were collected. The Whirl–Pak bags containing the larvae were then tightly closed to retain air before transporting to the laboratory as described by Rueda et al. [36], where they were identified and preserved.

### Preservation of adult mosquitoes and mosquito larvae

The trapped mosquitoes were processed as described by Tchouassi et al. [37]. The trapped mosquitoes were anaesthetized and killed using triethylamine while still in the trap. The mosquitoes were then sorted, counted, and put in Nunc tubes. The adult mosquitoes were then preserved in liquid nitrogen until when they were required for identification at Kenya Medical Research Institute (KEMRI), before processing them further for metal analysis. Similarly, the mosquito larvae were preserved as described by James-Pirri et al. [38]. Briefly, the mosquito larvae were retrieved from the Whirl–Pak bags and placed in hot water at a temperature of 87 °C for 50 s after which they were removed using a strainer. The larvae were then preserved in Dietrich’s solution and later transferred into 75% ethanol for further preservation until when they were required for identification and processing for metal analysis.

### Morphological identification of the trapped field mosquitoes

Both mosquito larvae and adults were identified using morphological features up to species level under a stereomicroscope. Appropriate mosquito taxonomic keys for the Sub-Sahara Africa and the East African region [39–41] were used.

### Laboratory rearing of mosquitoes

*Anopheles gambiae* s.s., Kisumu strain and *Aedes aegypti*, Mombasa strain laboratory colonized mosquitoes were reared in the laboratory at KEMRI, following the protocol described by Das et al. [42]. Mosquito rearing was carried out in the insectary that was maintained at a temperature ranging from 27 to 28 °C and approximately 80% humidity on a 12 h/12 h light and darkness cycle. Optimal larval concentrations were maintained to avoid possible effects of competition. Mosquito larvae were fed on finely ground *Sera Vipan staple diet ™* (Sera, Germany) while adults were offered a fresh 10% (w/v) glucose solution meal daily and fed on hamster (*Mesocricetus auratus*) as a source of blood meals for egg production. Mosquito larvae were reared in de-chlorinated tap water. De-chlorination of the tap water was achieved by allowing the tap water in a bucket to stand in the insectary chamber for at least 24 h. These laboratory-reared mosquitoes obtained served as a control in the current study to enable us to compare the levels of heavy metals in field trapped and laboratory mosquitoes.

### Preparation of the mosquito samples for metal analysis

Both the field and laboratory-reared mosquitoes were separately dried from an open room on brown papers, ground and then sieved to obtain a fine powder. The mosquito powder was then weighed, packaged in small new brown envelops and labeled appropriately to await metal analysis.

### Analysis of heavy metals for the different samples

The analysis of heavy metals was carried out at Mineral Laboratories, Bureau Veritas Commodities Ltd, Vancouver, Canada. The protocols included *aqua regia* digestion ultra-trace inductively coupled plasma mass spectroscopy (ICP-MS) for algae and mosquito samples; and ICP-MS (solutions > 0.1% TDS (total dissolved solids) for water samples as described by the American Herbal Products Association [43]. The digest solution was nebulized, and sample aerosols transferred to argon plasma. The high temperature plasma then produced ions, which were then introduced into the mass spectrometer. The mass spectrometer then sorted out the ions according to their mass-to-charge ration and finally, the ions were quantified with an electron multiplier detector. Certificates of analysis and quality control reports for all the samples analyzed were awarded by the Bureau Veritas, Canada.

### Data analysis

The statistical package for the social sciences (SPSS) version 20 for Windows at 5% level of significance was used for data analysis. Descriptive statistics involved computing mean, standard error (SE), and standard deviation (SD) for the different variables measured in wastewater, algae, and mosquito samples. One-way analysis of variance (ANOVA) was used, and Tukey’s & Games-Howell Post hoc tests were carried out to separate the means in the case of significant differences. Correlation analysis was carried out to establish the nature of relationship, and level of significance between concentrations of heavy metals in different samples. Pairwise correlation coefficients for the levels of selected heavy metals in wastewater, algae, and mosquito samples were also computed as described by Björklund et al. [44] and Benson et al. [45].

## Results

### Physico-chemical parameters of wastewater samples

The range for pH, temperature, total dissolved solids (TDS) and electrical conductivity (EC) of the wastewater samples were 7.28–8.78, 16.75–26.05 °C, 160.33–544.67 ppm, and 336.67–1134.33 µS/cm respectively (Table 1). All the wastewater samples obtained from the study area were alkaline, with those from Chief’s camp (B-1), Kartasi industries (F) and Sinai (G) sites being more alkaline at pH 8.13, 8.59 and 8.78, respectively. Samples of wastewater from open, shallow, and exposed drainage channel at Sinai (G) site had a temperature of 26.05 °C compared to samples from shaded channels and with a vegetation cover at Davis & Shirtliff sampling site (E) that had a temperature of 16.75 °C. Increased TDS corresponded to increased EC and vice versa. The TDS (ppm) of wastewater samples at sampling sites, Railways lower (C), Railways upper (D), and Sinai (G) were 562.00, 535.33 and 544.67 ppm; while the EC (µS/cm) of the wastewater samples in the same sites were 1134.33, 1072.33, and 1074.33 µS/cm respectively. Both TDS and EC recorded were above the recommended limits by WHO (Table 1).

**Table 1:** Range of variables measured compared to WHO standard limits for wastewater (effluent)

Variable measured (units)	Range	WHO limits	References
pH	7.28–8.78	6.5–8.5	Nazir et al. [46]
Temperature (°C)	16.75–26.05	20–32	Onuegbu et al. [47]
TDS (ppm or mg/l)	160.33–544.67	500	Onuegbu et al. [47]
Conductivity (µS/cm)	336.67–1134.33	400–600	Nazir et al. [46]

### Levels of heavy metals in samples of wastewater

The Pb levels were the highest ranging from 13.62 to 15.31 ppb or µg/l, followed by Ni (4.96–6.91 ppb or µg/l) and the lowest was Tl at 0.05 ppb or µg/l. The mean concentrations of the heavy metals in acidified wastewater samples were lowest for thallium and highest for lead in the order Tl < Hg < Cd < Cr < Ni < Pb (Table 2). Mean concentration of Cr (7.49 ± 2.12 ppb or µg/l) in wastewater samples that were not acidified was relatively higher than for the other elements studied (Table 2). The mean concentrations of Pb and Cr in acidified wastewater samples were above the limits set by WHO, US EPA and Kenya. The levels of Hg, Cd, and Ni in acidified wastewater samples were below the limits set by WHO and Kenya. The level of Hg in wastewater samples was above the US EPA limit which is set at 0.00003 ppm (0.03 ppb or µg/l). The mean concentration of thallium in wastewater samples was 0.04 ppb or µg/l.

**Table 2:** Mean concentration (ppb) of heavy metals in acidified and plain wastewater samples

Mean concentration	Heavy metals analyzed (ppb or µg/l)					
	Hg (0.1)^a^	Pb (0.1)	Cr (0.5)	Cd (0.05)	Tl (0.01)	Ni (0.2)
Wastewater samples						
(a) Acidified with HNO_3_ acid: Mean concentration ± SE	< 0.1 ± 0.00	15.31 ± 3.39	8.12 ± 5.40	0.09 ± 0.01	0.05 ± 0.009	4.96 ± 2.13
(b) Acidified with HCL acid: Mean concentration ± SE	< 0.1 ± 0.00	13.62 ± 2.91	3.08 ± 0.99	0.12 ± 0.02	0.05 ± 0.01	6.91 ± 2.69
(c) Plain (not acidified): Mean concentration ± SE	< 0.1 ± 0.00	0.18 ± 0.08	7.49 ± 2.12	< 0.05 ± 0.00	0.03 ± 0.009	0.57 ± 0.29
Standard limits for wastewater						
WHO [47–49]	1.0	10.0	5.0	3.0	–	20.0
US EPA [50]	0.03	6.0	5.0	10.0	–	200.0
Kenya [51]	5.0	10.0	5.0	10.0	–	300.0

^a^The value in bracket shows the method detection limit (MDL) measured in ppb (µg/l)

### Levels of the selected heavy metals in filamentous green algae

Filamentous green algae were sampled from 4 out of 8 (50%) sampling sites (Table 3). The difference in mean concentration of heavy metals in samples of wastewater and filamentous green algae collected from the same site was significant, F (11, 24) = 4.33, P < 0.05 (Table 4) at Kartasi site. Tukey post hoc test showed that the difference between the mean concentration of Cr in algae and concentrations of Hg, Pb, Cr, Cd, Tl and Ni in wastewater samples was significant (P = 0.006) at Kartasi site. The average heavy metal concentrations in filamentous green algae samples were between 500 to 5000 times more than the mean concentration of the same metals in wastewater samples in the same sampling site (Tables 2, 3, and 4). The mean concentrations of heavy metal in filamentous green algae were lowest for Hg and highest for Pb in the order Hg < Tl < Cd < Ni < Cr < Pb and ranged from 0.057 to 110.62 mg/kg (Table 3). The algae samples obtained from Railways Lower (D) and Davis & Shirtliff (E) sampling sites had a relatively higher level of heavy metals compared to those collected from Kartasi (F1a & Flb) sampling sites (Table 3). Concentrations of Hg, Pb, Cr, Cd and Tl were 1.93–2.75 times higher in room temperature dried algae samples than in lyophilized algae samples (Table 3). The Ni level was however higher in lyophilized algae samples than in the room temperature dried algae samples (Table 3).

**Table 3:** Concentration of heavy metal (mg/kg) in filamentous algae samples in three out of eight sites

Site of samples collection	Heavy metals that were analyzed (mg/kg)						
	Sample’s code	Hg (1.0)^a^	Pb (0.1)	Cr (0.5)	Cd (0.05)	Tl (0.01)	Ni (0.2)
Railways lower	D1a (room dried)	0.093	166.27	72.70	0.95	0.41	27.80
Davis & Shirtliff	E1a (room dried)	0.075	176.69	23.70	0.46	0.26	9.30
Kartasi	F1a (room dried)	0.040	65.54	15.30	0.22	0.22	7.70
Kartasi	F1b (freeze dried)	0.020	33.97	7.30	0.14	0.08	13.00
Mean concentration (ppm or mg/kg)		0.057 ± 0.02	110.62 ± 35.79	29.75 ± 14.71	0.44 ± 0.18	0.24 ± 0.07	14.45 ± 4.59

^a^The value in bracket shows the method detection limits (MDL) measured in mg/kg except for Hg which was in µg/kg. However, the data for Hg has been converted to mg/kg for consistency. Samples of algae from Kartasi site were in large quantity and therefore were divided into two portions, and one portion was freeze-dried (F1b) while the remaining portion was room temperature dried (F1a) before metal analysis

**Table 4:** Statistical comparison of levels of heavy metals in filamentous algae and wastewater at Kartasi sampling site

Site of samples collection	Concentration of heavy metals that were analyzed (mg/l or mg/kg)						
	Sample’s code	Hg (1.0)^a^	Pb (0.1)	Cr (0.5)	Cd (0.05)	Tl (0.01)	Ni (0.2)
Filamentous algae (mg/kg)							
Kartasi (sample 1)	F1a (room dried)	0.040	65.54	15.30	0.22	0.22	7.70
Kartasi (sample 2)	F1b (freeze dried)	0.020	33.97	7.30	0.14	0.08	13.00
Mean concentration ± SE		0.03 ± 0.01	49.76 ± 15.79	11.30 ± 4.00	0.18 ± 0.04	0.15 ± 0.07	10.35 ± 2.65
Wastewater (mg/l)							
Kartasi (sample 1)	F1	0.00010	0.0173	0.0014	0.00008	0.00003	0.0065
Kartasi (sample 2)	F2	0.00010	0.0106	0.0011	0.00007	0.00004	0.0036
Kartasi (sample 3)	F3	0.00010	0.0001	0.0065	0.00005	0.00001	0.0002
Mean concentration ± SE		0.0001 ± 0.00	0.0093 ± 0.005	0.0030 ± 0.002	0.0001 ± 0.00	0.00003 ± 0.00	0.0034 ± 0.001
Significance levels							
ANOVA test: F (11, 24) = 4.33, P < 0.05							

^a^The value in bracket shows the method detection limits (MDL) measured in mg/kg except for Hg which was in *µ*g/kg. However, the data for Hg has been converted to mg/kg for consistency

### Levels of heavy metals in mosquito samples

**Fig. 4:**
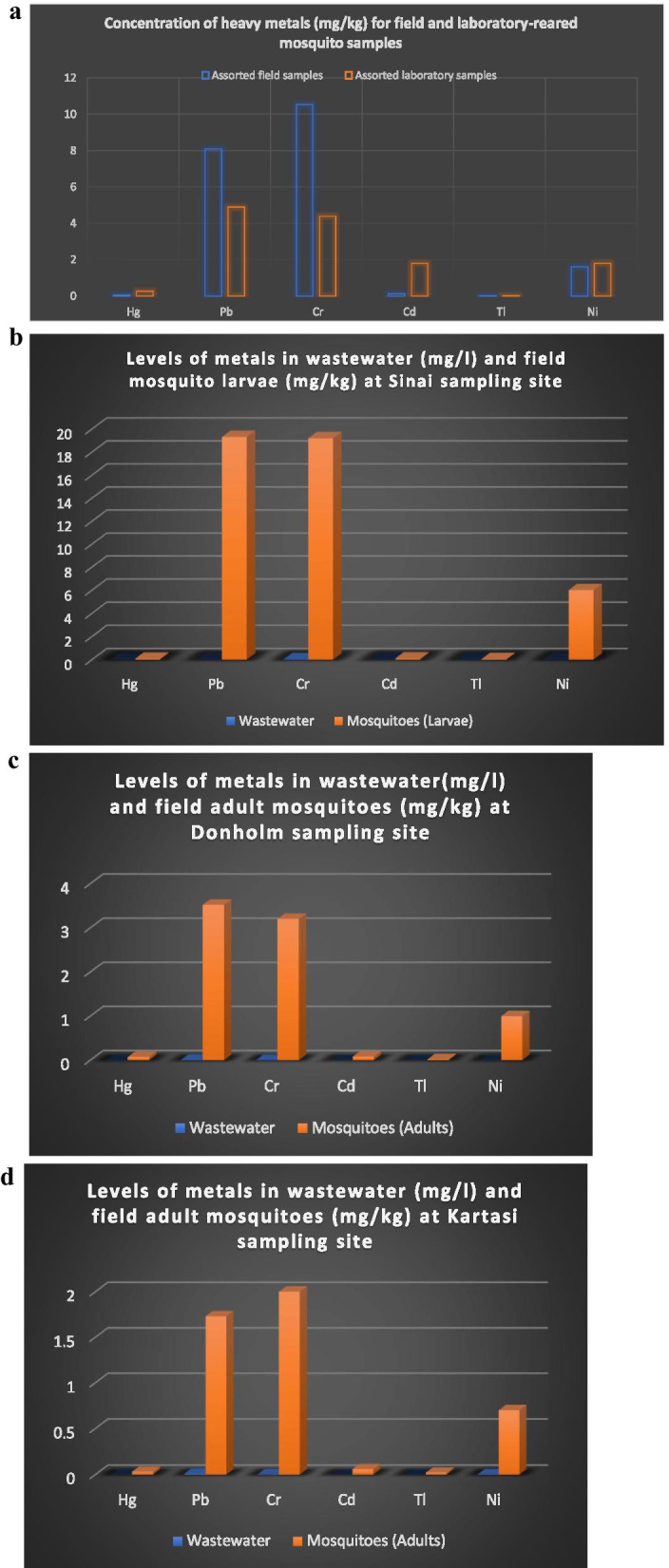
**a** Comparison of heavy metal concentration (mg/kg) in assorted field and assorted laboratory-reared mosquito samples. **b** Levels of metals in wastewater (mg/l) and field mosquito larvae (mg/kg) at Sinai sampling site. **c** Levels of metals in wastewater (mg/l) and field adult mosquitoes (mg/kg) at Donholm sampling site. **d** Levels of metals in wastewater (mg/l) and field adult mosquitoes (mg/kg) at Kartasi sampling site

Adult mosquitoes were trapped from sampling sites A, B, D, E, F, G and H while the mosquito larvae were collected from sites A, C, D, E and G. Assorted field mosquito samples were prepared from adult mosquitoes from sites A, B, D, E and G which were combined with mosquito larvae from sites A, C, D and E. Combining the field mosquito samples was necessary to meet the minimum sample weight of 0.5 g required for metal analysis protocol (VG 101-EXT) at Mineral Laboratories, Bureau Veritas Commodities Ltd, Vancouver, Canada. Similarly, assorted laboratory mosquito samples were made up of a mixture of laboratory reared adult Anopheles and Aedes mosquitoes and their larvae (Table 5). Concentrations of Hg, Pb, Cr, Cd, and Ni in adult Culex mosquitoes’ samples collected from Donholm site (H) was relatively higher than the means for the same elements at Kartasi site (F) as shown in Table 5. Similarly, the mean concentrations of Hg, Pb, Cr, Cd, Tl, and Ni in mosquito larvae samples collected from Sinai site (G) were relatively higher compared to the means of the same elements in adult mosquitoes trapped at Kartasi and Donholm sites (Table 5). The mean concentration of heavy metals in field mosquitoes’ samples was lowest for Tl and highest for Cr giving the order Tl < Hg < Cd < Ni < Pb < Cr while that for the laboratory reared mosquito samples was Tl < Hg < Cd & Ni < Cr < Pb (Table 5). The mean concentration of Pb, Cr, Tl, and Ni in assorted field mosquito samples were 1.3–2.4 times more than the mean concentration in the assorted laboratory reared mosquito samples. The mean concentrations for Hg (0.26 mg/kg) and Cd (1.8 mg/kg) in assorted laboratory-reared field mosquitoes were 4.4 and 20 times more respectively than in assorted field mosquitoes which were at 0.059 mg/kg (Hg) and 0.09 mg/kg (Cd) as shown in Fig. 4a and Table 5. The mean concentration of Pb, Cr, Cd, Tl, and Ni at Kartasi sampling site, in filamentous algae samples was 3–29 times higher than in assorted field mosquito samples (Tables 3 and 5). The level of Tl was below the method detectable level which was set at 0.02 mg/kg in both the assorted field and laboratory reared mosquito samples (Table 5).

**Table 5:** Comparison of heavy metal concentrations (mg/kg) in laboratory- reared and field mosquitoes

Site of sample collection/assortment	Heavy metals analyzed and their concentration (mg/kg)						
	Sample’s code	Hg (1.0)^a^	Pb (0.01)	Cr (0.1)	Cd (0.01)	Tl (0.02)	Ni (0.1)
Field samples							
Sinai (Mean)	G-m (Cx: larvae)	0.084	19.31	19.20	0.10	0.06	6.00
Kartasi	F-m (Cx: adults)	0.031	1.73	2.00	0.06	< 0.02	0.70
Donholm	H-m (Cx: adults)	0.072	3.52	3.20	0.08	< 0.02	1.00
Assorted field samples^b^	As-f (Cx: adults, larvae)	0.048	8.09	17.70	0.12	< 0.02	1.60
Mean ± SE (ppm)		0.059 ± 0.01	8.16 ± 3.95	10.53 ± .59	0.09 ± 0.01	0.03 ± 0.01	2.33 ± 1.24
Control samples							
Assorted laboratory samples	As-l^c^	0.260	4.89	4.40	1.80	< 0.02	1.80

^a^The digit in bracket shows the method detection limits (MDL) measured in mg/kg except for Hg which was in µg/kg. However, the data for Hg was converted into mg/kg for consistency

^b^Assorted field samples implies a mixture of adult mosquitoes and larvae trapped from the field

^c^Assorted laboratory samples implies a mixture of laboratory reared adult Anopheles and Aedes mosquitoes and their larvae

The mean concentration of Pb, Cr, and Ni in both assorted field and assorted laboratory-reared mosquitoes’ samples ranged from 2.33 to 10.53 ppm or mg/kg. (Table 5). The level of Hg in both assorted field and assorted laboratory-reared mosquitoes’ samples ranged from 0.06 to 0.26 mg/kg. Similarly, the mean concentration of Cd in assorted laboratory-reared mosquito samples was 1.8 mg/kg. While the mean Cd level in assorted field mosquito samples was 0.09 mg/kg (Table 5).

Levels of metal pollutants in the wastewater samples were compared with the same elements in field mosquito samples at the same sampling site. It was established that the concentration of Pb, Cr, and Ni was distinctively higher in mosquito samples than in wastewater samples obtained from the same sampling site (Fig. 4b–d).

### Correlation of the heavy metal levels in wastewater, algae, and mosquito samples

A positive correlation was noted between Pb & Hg (r (2) = 0.957, p = 0.043); Cd & Cr (r (2) = 0.985, p = 0.015); and Tl & Hg (r (2) = 0.946, p = 0.054) in algae samples (Table 6). Similarly, Tl & Cd in wastewater samples correlated positively (r (7) = 0.631, p = 0.069) (Table 6). The strong positive correlations between specific pairs of elements may have suggested uniformity of the elements in wastewater and filamentous algae in terms of their source and accumulation behavior [52]. Strong negative correlations were noted for Pb (wastewater) & Hg (algae) where r (2) = − 0.921, p = 0.079; and for Pb (algae) & Pb (wastewater) where r (2) = − 0.974, p = 0.026 (Table 6).

**Table 6:** Inter-elemental correlation of heavy metal concentrations in samples of wastewater and filamentous green algae

Pairs correlated	Correlation co-efficient (r value)	P value
Pb (algae^a^) & Hg (algae)	0.957	0.043*
Pb (algae) & Pb (wastewater)	− 0.974	0.026*
Pb (wastewater) & Hg (algae)	− 0.921	0.079
Cd (algae) & Cr (algae)	0.985	0.015*
Cd (algae) & Hg (algae)	0.928	0.072
Tl (wastewater) & Cd (wastewater)	0.631	0.069
Tl (algae) & Hg (algae)	0.946	0.054
Tl (algae) & Cr (algae)	0.924	0.076
Tl (algae) & Cd (algae)	0.939	0.061

*Correlation significance at 0.05 level (2 tailed)

^a^Type of sample analyzed is placed in brackets

Table 7 shows inter-elemental correlation of the mean concentration of pairs of heavy metals in wastewater samples obtained from Sinai site (G). Pairs of elements such as Cd & Pb; and Ni & Pb correlated positively and significantly (P < 0.05) in wastewater. Similarly, Cd & Pb; and Tl & Pb in samples of mosquito larvae (Table 7) trapped from wastewater at Sinai showed strong negative significant correlation (P < 0.01) while Tl & Cd showed positive significant correlation (P < 0.01) as shown in Table 7.

**Table 7:** Correlation (r values) of heavy metal concentrations in wastewater and mosquito larvae samples from Sinai site

	Hg	Pb	Cr	Cd	Tl	Ni
Wastewater						
Hg	–^a^					
Pb	–	1				
Cr	–	− 0.820	1			
Cd	–	0.998*	− 0.784	1		
Tl	–	0.453	− 0.881	0.397	1	
Ni	–	0.998*	− 0.858	0.992	0.513	1
Mosquito larvae						
Hg	1					
Pb	0.009	1				
Cr	− 0.225	0.972	1			
Cd	− 0.022	− 1.000**	− 0.969	1		
Tl	− 0.022	− 1.000**	− 0.969	1.000**	1	
Ni	− 0.418	− 0.912	− 0.792	0.918	0.918	1

*Correlation significance at the 0.05 level (2 tailed)

**Correlation significance at the 0.01 level (2-tailed)

^a^Correlation could not be computed because one of the variables was constant (level of Hg was < 0.1 ppb throughout)

## Discussion

The mean concentration of Pb (14.47 ppm) and Cr (5.6 ppm) in acidified wastewater samples from Nairobi industrial area were above the limits set by WHO, US EPA and Kenya for wastewater (effluent). This observation was in line with a study carried out by Kaluli et al. [53] who observed low levels (below WHO, US EPA, and Kenyan limits for wastewater) of Pb and Cr in wastewater samples obtained outside Nairobi industrial area at Kibera informal settlements. In the current study, the industrial effluents which often contain heavy metals, may have found their way into the open drainage channels hence contributing to the increased levels of Pb and Cr in the samples of wastewater analyzed. This agreed with a study carried out by Njuguna and his colleagues [31] who linked the high concentration of Pb, Cr and Ni in Ngong river (Fig. 1) to industrial effluent in Nairobi industrial area. The average levels of Pb, Cr, Cd, and Ni in assorted field mosquito samples in our study ranged from 0.09 to 10.53 mg/kg. This was in line with an observation made by Kitvatanachai et al. [12] in their study, whereby *Culex* mosquito larvae trapped from larval habitats near a Lead factory in Thailand, had a high Pb mean concentration of 11.76 µg/g. The concentration of the elements studied were relatively higher in the filamentous algae and least in the wastewater. Environmental contaminants bioaccumulate and bio magnify in the food chains, increasing their concentration along a food chain [54]. This was not the case in the current study because the concentration of heavy metals was higher in filamentous green algae than in mosquitoes, which were presumed to be the consumers of these algae. Further investigation on the factors that influence bioaccumulation of heavy metals in mosquitoes in aquatic ecosystems is therefore necessary.

According to Azam et al. [55], insects are the dominant invertebrate faunal group that has been used in biomonitoring and bio assessment studies. This is because insects are highly diverse and able to adapt to a wide range of habitats, hence accomplishing many ecological roles in the ecosystems [56]. Insects are abundant and they possess diverse morphologies and functions which enable them to display unique biochemical and genetical responses after their exposure to environmental changes including pollutions. In the current study, field urban mosquitoes were evaluated for their possible role of bio-indication for heavy metal pollution in wastewater since they frequently encounter such water in open drainage channels when accomplishing their life processes including feeding and reproducing. The results from our study showed that the field urban mosquito samples, majority of which belonged to *Culex* species as previously reported [13], had high levels of Pb and Cr compared to the laboratory-reared *Anopheles* and *Aedes* control mosquitoes that were reared in KEMRI—Nairobi, Kenya. Similarly, concentration of Pb, Cr, and Ni was distinctively higher in mosquito samples than in wastewater samples from the same site. This may have implied that the mosquitoes that bred in contaminated wastewater may have absorbed and accumulated the elements into their body tissues. Previous studies have shown that *Culex* mosquito larvae can be tools for natural biomonitoring of heavy metals since they are among the first in the food chain to be exposed to the heavy metal pollutants [27, 57]. It was also observed in the current study that the laboratory-reared mosquito samples had a slightly higher level of metals including Hg, Cd & Ni when compared to the field mosquito samples (Table 5). This could have been probably attributed to the rearing processes, equipment used, insectary, insect feed, and routine procedures in the rooms adjacent to the insectary where the rearing of mosquitoes took place. According to van der Fels-Klerx et al. [58], insects can become exposed to chemical hazards from the substrate used to grow them. Some of the heavy metals are known to escape into the air as tiny particulates [59] which would then easily contaminate the insectary and the mosquitoes being reared. Laboratories have been associated with increased concentration of specific pollutants depending on the nature of experiments that are being conducted [60]. In a study on indoor air quality in research laboratories, Valavanidis and Vatista [61] established that respirable suspended particulates (RSP) reached 700 µg/m^3^ in spring and summer period. Similarly, Rumchev et al. [62] in their study on indoor air quality in 15 university laboratories established that the particulate matter (PM_2.5_ and PM_10_) were significantly high in Chemistry, Engineering and Biology laboratories. Suspended particulates in the air may include black carbon, heavy metals, spores, dust, pollen grains, liquid aerosols among others, and they tend to be in large quantities in heavily polluted areas and premises.

The mean concentration of heavy metals in the samples studied were generally higher in filamentous algae, followed by mosquito, and least in wastewater. For instance, at Kartasi sampling site, the mean concentration of Pb, Cr, Cd, Tl, and Ni in filamentous algae samples was 3–29 times higher than in assorted field mosquito samples. This observation was in line with a previous study carried out by Kitvatanachai et al. [12] which showed that the levels of Pb was higher in *Cx. quinquefasciatus* than in wastewater from the factories and the areas close to the factories. Aquatic insects accumulate heavy metals in their bodies from contaminated aquatic ecosystems because they become exposed during their vital developmental stages and processes including embryogenesis, larval development, and pupation [63, 64]. The emerging and surviving imagoes of the aquatic insects are therefore likely to have elevated levels of heavy metals in their bodies after exposure. In our current studies, field assorted mosquitoes had high concentration of Pb and Cr when compared to the assorted laboratory-reared mosquitoes. The sampled field mosquitoes may have gained heavy metals from the contaminated wastewaters through diffusion or by feeding on contaminated materials. Environmental pollution, a human activity, may therefore compel the mosquitoes to undergo evolution and make them to survive in modified habitats [65]. According to Brooks et al. [66], evolutionary success of an organism is its capacity to cope up with environmental changes over short and long periods, hence extending its survival. It is worth noting though, from previous studies that the process of metamorphosis can be a survival challenge for aquatic insects in metal contaminated aquatic ecosystems because the larvae become exposed to extra stress that enhance the mortality of the imagoes [67]. The mosquitoes in urban areas, majority of which are *Culex pipiens* [13] can however breed in polluted wastewater, although when exposed to increased specific heavy metal concentration, their breeding potential is reduced [68]. According to Dom et al. [69], the Aedes mosquitoes, the key dengue vectors appear to develop adaptations to cope with increased heavy metal concentration in polluted waters. The mosquitoes from urban areas that can breed and survive in polluted waters especially in crowded areas are therefore a health hazard because they can serve as vectors of infectious diseases as well as pollutants contaminated insects. In a previous study, the potential of *Culex* mosquitoes in transferring pollutants in the environment has been demonstrated [70]. The current study established that the mean levels of Pb, Cr, and Ni were higher in assorted field mosquito samples than in assorted laboratory mosquito samples. This was in line with a previous study that reported an increased Pb levels in *Cx. quinquefasciatus* mosquito larvae that were obtained from Pb-contaminated wastewater [12].

From previous studies, algae belonging to the genus *Spirogyra* have a potential of absorbing heavy metal from contaminated water [71, 72]. Our current study established that the mean concentration of heavy metals analysed in filamentous green algae ranged from 0.057 to 110.62 mg/kg, and this concentration was 500–5000 times more than the mean concentration of the same metals in the wastewater samples collected from the same site. The green algae *Spirogyra* species have the potential of adsorbing 10–40 mg/g of Pb^2+^ ions from aqueous solutions containing the ions [73]. Vetrivel et al. [72] in their study observed that algae of the genus *Spirogyra* are efficient biosorbent material for heavy metal removal in coal mine water. According to Sunish and Reuben [74], filamentous algae in the mosquito breeding water have nutritive value necessary for mosquito development and adult emergence. In a study that involved the examination of the gut contents in mosquito larvae, Charles et al. [75], were able to identify *Spirogyra* algae in the gut of *Anopheles stephensi*. Tuno et al. [15] established that the presence of filamentous green algae (*Spirogyra* sp.) was significantly correlated with animal (which included mosquito larvae) assemblage in the water. Therefore, when the mosquito larvae feed on heavy metal contaminated filamentous algae, the heavy metals may get transferred into their tissues. Feeding process is one of the main pathways through which aquatic invertebrates obtain metals from their surroundings [76, 77]. Our study clearly illustrates occurrence of bioaccumulation of heavy metals in the mosquitoes and aquatic filamentous algae inhabiting contaminated wastewater in open drainage channels in Nairobi industrial area, Kenya. Previous studies have established that contaminated wastewater could lead to a build-up of heavy metals in soils, food crops and macrophytes [31, 78]. According to Hamidian et al. [79], the algae of the genus *Spirogyra* are suitable for biomonitoring purposes because they are capable of accumulating heavy metals from polluted water. Use of algae for environmental biomonitoring can be advantageous and suitable because algae are spatially dense, easy to sample where available and store [28]. Similarly, mosquitoes are advantageous in biomonitoring because they can breed rapidly in stagnant water and are easy to sample, especially the larvae.

Inter-elemental analysis of the metals in the algae samples revealed statistical correlations of Pb & Hg, Cd & Cr, and Tl & Hg. These correlations suggested that the pairs of the metals may have had a common source, most likely the industries whose wastes were draining into the open drainage channels in the study area. It was observed in the current study that the wastewater in the open drainage channels were finally flowing into Ngong river which flows through Nairobi industrial area. Such industries probably were releasing specific wastes that were rich in certain elements that had a similar accumulation behavior, hence a positive correlation of such elements. This may require further investigation to verify. This explanation was in line with previous studies carried out in South Africa, Nigeria, and Pakistan [52, 80, 81]. The significant correlation coefficients between pairs of metals in samples of wastewater, filamentous algae and mosquito larvae may have suggested that the sources of the heavy metal pollution in the study area was mainly anthropogenic. Strong and significant negative correlations of specific elements including Pb (wastewater) and Hg (Algae); Pb (algae) and Pb (wastewater), may require further investigation on the factors that influence their uptake by aquatic algae and mosquito larvae in wastewater. Previous reports attributes negative correlation of elements in aquatic ecosystem to their solubility behavior [31].

Our current study raises a few public health concerns such as, people can easily become exposed to heavy metal pollutants when clearing and unblocking the open drainage channels when they clog in the study area. Prolonged heavy metal exposure can lead to serious toxicity and exposure to potential carcinogenic agents in humans [82]. The heavy metal contaminated wastewater pollutes the surface runoffs after the rains, which then spread the pollutants into the residential areas, soils, crops, and public places. Contaminated wastewater in the drainage channels may overflow and spread onto the highways during the heavy rains hence exposing the road users to the pollutants. The mosquitoes that breed successfully from contaminated wastewater may accumulate heavy metals in their bodies with time through direct diffusion of such metals into their bodies or by ingesting heavy metal contaminated plant materials that includes algae. Such mosquitoes may therefore serve as both disease vectors as well as insects contaminated with pollutants. Studies to verify whether mosquitoes with elevated heavy metals in their tissues can spread such elements to their hosts are however lacking. Such a study can involve comparing the levels of heavy metals in salivary glands of mosquitoes that are exposed and those not exposed to heavy metals. Previous reports indicate that use of microalgae in wastewater treatment and biofuel production is on the rise [83, 84]. The current study has shown that algae present in contaminated raw wastewater absorbs and accumulates the pollutants, in this case heavy metals. This observation was in line with a previous report which highlighted on the use of algae in absorption of contaminants like ammonium compounds, nitrates, and heavy metals present in raw wastewater [85]. In the current study, the levels of heavy metals in algae were higher than in wastewater due to bioaccumulation. Therefore, use of appropriate and effective methods is key when harvesting microalgae grown during wastewater treatment, for eventual post-harvest processes that include biofuel and animal feed production among other uses [85–87].

## Conclusion

The samples of wastewater, filamentous green algae and mosquitoes obtained from open drainage channels in Nairobi industrial area contained heavy metals. The mean concentration of Pb, Cr, and Ni were relatively higher than those of Tl, Hg, and Cd in all the samples analyzed. The concentration of Pb, Cr, Ni, and Cd in wastewater were above the limits set by WHO, Kenya and US EPA for wastewater (effluents) however, the level of Hg in wastewater was not. Further investigation to identify the specific factory discharges that contaminate the drainage channels with heavy metals may be necessary. The levels of Pb, Cr, Tl, and Ni in assorted field mosquito samples was relatively higher than in assorted laboratory-reared mosquito samples. The mean concentration of heavy metals in field mosquito samples followed an ascending order of Tl < Hg < Cd < Ni < Pb < Cr. The concentration of Pb, Cr, and Ni was distinctively higher in field mosquito samples than in wastewater samples at the same site. Positive correlations were noted for Pb & Hg (r = 0.957), Cd & Cr (r = 0.985) and Tl and Hg (r = 0.946) in algae samples suggesting a probable association of the elements in terms of their source and accumulation behavior. Similarly negative correlations were noted for Pb (wastewater) & Hg (algae), r = − 0.921, among others and which may require further investigation on factors that influence their uptake by algae and mosquito larvae in wastewater. Both filamentous algae and urban mosquitoes growing and breeding respectively in contaminated wastewater in open drainage channels were bio-accumulating the heavy metals and therefore have the potential of being used for heavy metal pollution biomonitoring. However, there is need to establish evidence to support that the mosquitoes breeding in the open drainage channels were feeding on the filamentous algae to verify the flow of the elements in the aquatic food chains. In addition, there is need for efficient wastewater management and treatment in Nairobi industrial area to minimize exposure of the vulnerable population living in the neighborhood to the hazardous contaminants. Strict environmental and public health policies should be formulated, adopted, and made to work to manage industrial effluents effectively. Public awareness on the health risks associated with untreated wastewater should be done to the residents living in the informal settlements in the study area. Safety measures, equipment and apparatus should be availed whenever the municipal workers and youth groups are unclogging and cleaning up the open drainage channels in the study area. The possibility of urban mosquitoes transferring the heavy metals to the urban population from the contaminated wastewater should be investigated further. Since this study was carried out during the dry period in Nairobi, we recommend the collection of the same samples be repeated during the long and short rains periods, and their heavy metal contents determined.

## Limitations

We acknowledge the limitations of the current study which included: limited samples of filamentous green algae from the sampling sites and the challenge faced in obtaining adequate powdered mosquito samples for adults and larvae separately for metal analysis, hence forcing us to prepare assorted (mixed) mosquito samples for metal analysis.

## Data Availability

All the datasets generated and/or analyzed during the current study are not publicly available but are available from the corresponding author on reasonable request.

## Abbreviations

ANOVA: Analysis of Variance
AHPA: The American Herbal Products Association
CDC: Centers for Disease Control and Prevention
EC: Electrical conductivity
EMCA: Environmental Management and Coordination Act
ICP-MS: Inductively Coupled Plasma Mass Spectroscopy
KEMRI: Kenya Medical Research Institute
MDL: Method detection limit
NACOSTI: National Commission for Science, Technology, and Innovation
RSP: Respirable suspended particulates
SD: Standard deviation
SE: Standard error
SPSS: Statistical Package for the Social Sciences
TDS: Total dissolved solids
US EPA: United States Environmental Protection Agency
WHO: World Health Organization
